# Small EVs-Associated DNA as Complementary Biomarker to Circulating Tumor DNA in Plasma of Metastatic Colorectal Cancer Patients

**DOI:** 10.3390/ph14020128

**Published:** 2021-02-06

**Authors:** Silvia Galbiati, Francesco Damin, Dario Brambilla, Lucia Ferraro, Nadia Soriani, Anna M. Ferretti, Valentina Burgio, Monica Ronzoni, Riccardo Vago, Laura Sola, Marcella Chiari

**Affiliations:** 1 Complications of Diabetes Units, Diabetes Research Institute, IRCCS San Raffaele Scientific Institute, 20132 Milan, Italy; 2 Istituto di Scienze e Tecnologie Chimiche “Giulio Natta” SCITEC CNR, 20131 Milan, Italy; dario.brambilla@scitec.cnr.it (D.B.); lucia.ferraro@scitec.cnr.it (L.F.); anna.ferretti@scitec.cnr.it (A.M.F.); laura.sola@scitec.cnr.it (L.S.); marcella.chiari@scitec.cnr.it (M.C.); 3 Unit of Genomic for the Diagnosis of Human Pathologies, Division of Genetics and Cell Biology, IRCCS San Raffaele Scientific Institute, 20132 Milan, Italy; soriani.nadia@hsr.it; 4 Dipartimento di Oncologia Medica, IRCCS Ospedale San Raffaele, 20132 Milan, Italy; burgio.valentina@hsr.it (V.B.); ronzoni.monica@hsr.it (M.R.); 5 Urological Research Institute, Division of Experimental Oncology, IRCCS Ospedale San Raffaele, 20132 Milan, Italy; vago.riccardo@hsr.it; 6 Università Vita-Salute San Raffaele, 20132 Milano, Italy

**Keywords:** exosomes, extracellular vesicles, liquid biopsy, cancer biomarkers, ddPCR, microarray

## Abstract

It is widely accepted that assessing circular tumor DNA (ctDNA) in the plasma of cancer patients is a promising practice to evaluate somatic mutations from solid tumors noninvasively. Recently, it was reported that isolation of extracellular vesicles improves the detection of mutant DNA from plasma in metastatic patients; however, no consensus on the presence of dsDNA in exosomes has been reached yet. We analyzed small extracellular vesicle (sEV)-associated DNA of eleven metastatic colorectal cancer (mCRC) patients and compared the results obtained by microarray and droplet digital PCR (ddPCR) to those reported on the ctDNA fraction. We detected the same mutations found in tissue biopsies and ctDNA in all samples but, unexpectedly, in one sample, we found a *KRAS* mutation that was not identified either in ctDNA or tissue biopsy. Furthermore, to assess the exact location of sEV-associated DNA (outside or inside the vesicle), we treated with DNase I sEVs isolated with three different methodologies. We found that the DNA inside the vesicles is only a small fraction of that surrounding the vesicles. Its amount seems to correlate with the total amount of circulating tumor DNA. The results obtained in our experimental setting suggest that integrating ctDNA and sEV-associated DNA in mCRC patient management could provide a complete real-time assessment of the cancer mutation status.

## 1. Introduction

Circulating tumor DNA (ctDNA) belongs to the pool of the total circulating free-DNA (cfDNA) in blood, but it primarily derives from tumors. ctDNA provides real-time molecular information to monitor treatment response and relapse as it contains genetic alteration of both primary and metastatic lesions, such as point mutations, copy number variations and insertions/deletions. The mechanism of the release of ctDNA is not completely understood; it derives from apoptotic or necrotic cells as well as from living cells through a mechanism of active secretion.

It has been demonstrated that tumor cells have to shed a larger number of small extracellular vesicles (sEVs) than normal cells to elude the immune-systems or prepare a metastatic niche [1]. Therefore, sEVs are promising biomarkers in several tumors [2].

The population of sEVs comprises diverse subpopulations (mainly exosomes, microvesicles, and apoptotic bodies) that differ in size, morphology, composition, or biogenic mechanisms [3].

In particular, exosomes are 40–120 nm nanovesicles of endosomal origin secreted by cells into bodily fluids. Their specific cargo (metabolites, nucleic acids, and proteins) and membrane proteins are a signature that reflects the cell of origin [4,5]. Exosomes are now recognized as a critical component in the complex communication between cells in tumor progression and metastasis and are increasingly gaining attention as elements that could provide enormous opportunities for both diagnostics and therapy [6].

Few and controversial studies have been conducted until now on DNA content in extracellular vesicles and specifically on the class of particles called exosomes.

In 2014, Thakur et al. demonstrated for the first time that the majority of DNA associated with tumor exosomes is double-stranded (dsDNA) and represents the whole genomic DNA [4]. This finding highlights the significant translational potential of exosomal DNA as a tumor circulating biomarker in clinical settings for several reasons: its protection and thus inherent stability within exosomes; the possibility to isolate or enrich tumor-derived exosomes in complex plasma samples via exosomal surface markers; and its easy and fast preparation [4]. In the same year, Lee et al. demonstrated that oncogenic ras-driven cancer cell vesiculation leads to the emission of double-stranded DNA capable of interacting with target cells [7]. Subsequently, various authors highlighted the importance of circulating exosomal DNA for a rapid, low-cost identification of cancer driving mutations in pancreatic and prostate cancer patients [8,9,10]. More recently, a reassessment of exosome composition by Jeppesen and colleagues established that small extracellular vesicles are not vehicles of active DNA release [11]. However, this paper’s most significant criticism concerns the number of exosomes used in the analytical assays, which might have led to ambiguous conclusions [12]. Zocco et al. [13], in disagreement with Jeppesen, suggested that about 90% of the isolated mutant DNA is out of the sEVs (associated with the outer surface of the EV membrane or independently co-purified within protein aggregates). They found that only about 10% of DNA was detected inside the exosomes, after treatment with DNase I. However, this internal DNA was useful to improve the detection of circulating mutant DNA in melanoma patients with low tumor mutation burden.

In the literature, there is no consensus on the presence of dsDNA in exosomes, and the topic is still open for discussion.

Our group is actively involved in developing methods to detect point mutation in liquid biopsy [14,15,16]. The assessment of the best biomarker source among circulating DNA, DNA externally associated or contained in sEVs, both alone or combined, could be of great importance in diagnostics. To shed light on this topic, we investigated the presence of DNA in sEV fractions of eleven metastatic colorectal cancer (mCRC) patients with two different methodologies (microarray and droplet digital PCR) and compared the results with those previously obtained analyzing ctDNA in the same samples [14].

Our data confirm the presence of *KRAS* and *BRAF* mutations in cell-free circulating and sEV-associated DNA, in particular a higher concentration and fractional abundance is detected in ctDNA.

Surprisingly, we identified in one patient a mutation in the DNA associated with sEVs that was not detected in ctDNA. We hypothesize that mutated sequence in cell free DNA (cfDNA) was not concentrated enough to be revealed by the techniques used for the analysis despite their excellent sensitivity.

Moreover, our data confirm a significant reduction in the amount of DNA found in sEVs after treatment with DNase I. In some cases, the drop is so substantial that the DNA detection becomes challenging unless the total amount of circulating DNA is high. A profound analysis of the results provided by the analysis of DNA from different sources proved to be useful.

Our results suggest that besides ctDNA, the tumor-derived fragmented DNA in the bloodstream that is not associated with cells, sEV-associated DNA fractions can help to identify and monitor mutations in mCRC patients.

## 2. Results

### 2.1. sEV-Associated DNA Analysis in mCRC Patients

Recently, we have introduced a new microarray approach to detect point mutations in the ctDNA of patients with metastatic colorectal cancer [14]. Droplet digital PCR (ddPCR) confirmed the mutation’s assignment of microarray and assessed their fractional abundance. Using eleven samples of the cohort reported in reference [14], we investigate which of the different tumor-driven component—ctDNA, DNA internally or externally associated with exosomes—should be used to determine tumor-specific molecular alterations and the differences in the information provided. Taking the genotype of ctDNA performed in [14] as a reference, we first carried out mutational analysis on the sEV-associated DNA of matched plasma samples by microarray to identify the mutation, and then ddPCR analysis to determine the fractional abundance.

**Figure 1:**
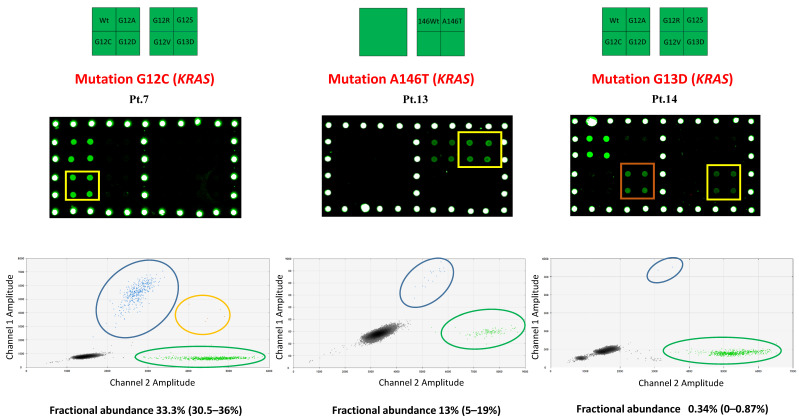
Analysis of sEV-associated DNA isolated from plasma of metastatic colorectal cancer (mCRC) patients. Upper panel: Schematic representation of the spotted barcode probe array; middle panel: Cy3 fluorescence images of the analysis of sEV DNA from plasma of patient 7, 13 and 14. The barcode probes corresponding to KRAS G12C, KRAS A146T, and KRAS G13D as attended for these patients are highlighted in yellow. In orange is highlighted the KRAS G12D mutation that was not detectable in ctDNA [14]. Lower panel: 2-D fluorescence amplitude plot generated by QuantaSoft™ software from plasma EVs of patient 7, 13 and 14 and respective fractional abundance of the mutated KRAS allele. Mutant sequences are clustered in the top center with high FAM fluorescent intensities (blue dots in the blue circle), wild-type sequences are clustered in lower right corner with high HEX fluorescent intensities (green dots in the green circle), while mutant plus wild-type sequences are clustered in the upper right corner (orange dots in the orange circle). The black cluster on the plot represents the negative droplets.

The results are reported in Table 1. For each sample, the type of mutation and fractional abundance found in ctDNA and sEV-associated DNA, as determined by microarray and ddPCR, are reported. The microarray analysis of the samples led to an unexpected result. Figure 1 shows microarray fluorescence images and ddPCR graphical results on three patient plasma samples with *KRAS* G12C (patient n. 7), *KRAS* A146T (patient n. 13), and *KRAS* G13D (patient n. 14) mutations. In the latter case, as shown in Figure 1, the microarray methodology identified the presence of two mutations: the mutation *KRAS* G13D, in agreement with the results of tissue biopsy and ctDNA analysis, but also the mutation *KRAS* G12D, which was not detected previously, either in ctDNA or in tissue biopsy (see 11).

### 2.2. sEV-Associated DNA Analysis after DNase I Treatment in mCRC Patients

**Figure 2:**
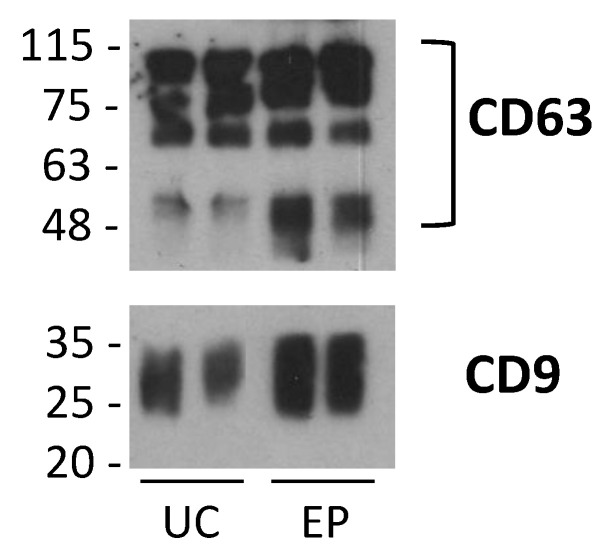
Western blot analysis of plasma EVs isolated from a patient by ultracentrifugation (UC) or Exosome Precipitation kit (EP) with anti-CD9 and anti-CD63 antibodies. The bracket indicates the typical CD63 separation, the smear is likely due to high degree of glycosylation.

CtDNA and DNA extracted from sEVs obtained with three different methodologies (ultracentrifugation, precipitation, and immunoprecipitation) belonging to patient n. 22 were analyzed by ddPCR. Western blotting was used to confirm the presence of EV transmembrane protein (tetraspanins CD63 and CD9) before starting with molecular analysis (Figure 2). We report only WB of ultracentrifuged and precipitated EVs, since the EV solution purified by immunoaffinity contains the antibodies used for the capturing that co-migrate with EV-associated proteins.

The immunoprecipitation approach we adopted in this work was recently introduced by our group [17]. It is based on the so-called DNA directed immobilization (DDI) strategy to anchor the anti-tetraspanin anti CD63 antibody on magnetic particles through a DNA-linker. The antibody is tagged with an oligonucleotide complementary to the one immobilized on the beads.

To release the sEVs, the DNA-linker is cleaved by DNase I. This immunoprecipitation approach implies that the DNA surrounding the vesicles is removed at the end of the separation. The results of the mutational analysis on exosomes obtained with the three purification methods are reported in Table 2. A significant reduction in the copies/number was seen in the sample subjected to DNase I treatment (immunoprecipitation approach). This finding seems to confirm the thesis that the majority of DNA is bound outside the vesicle, probably attached to the lipidic membrane by electrostatic interaction or to DNA binding proteins.

To confirm the significant reduction in DNA in the sEVs treated with DNase I, from 294 or 574 (by ultracentrifugation or precipitation, respectively) to 22 copies, we determined in parallel the content of DNA in sEVs isolated from a plasma sample by ultracentrifugation and precipitation before and after the DNase I treatment and by immunoprecipitation with DNase I sEVs release.

DNA extracted from purified sEVs in patient n. 2 was analyzed by ddPCR, specifically looking for the *BRAF* V600E mutation. The supernatant fraction (no DNase I) of the ultra-centrifuged sample were also analyzed in duplicate as reported in Table 3. For these assays, we selected in particular the patient n. 22 and n. 2 for the volume of the plasma available to perform in parallel several analyses. They also represented samples with different fractional abundance, very high for patient n. 22 (80%) and lower for patient n. 2 (15%).

**Figure 3:**
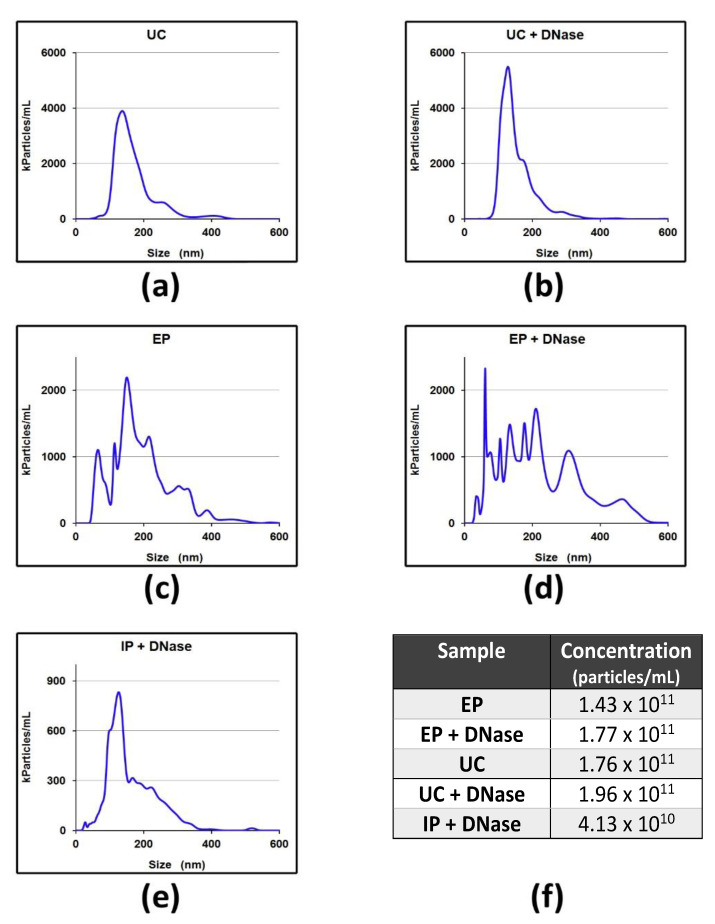
Nanoparticle tracking analysis of sEVs separated from plasma using ultracentrifugation (UC) (**a**), the Exosome Precipitation kit (EP) (**c**) or DNA-directed immunoprecipitation (IP) (**e**). Samples deriving from EP and UC were further treated with DNase I and particle size after enzymatic treatment are show in (**b**,**d**), respectively. sEV concentration, reported as particles/mL, is also shown (**f**).

The results in Table 3 unequivocally show that the sample treated with DNase I presents a significant reduction in the concentration of DNA (about 10 times less). This reduction is not due to a loss of cargo. As shown in Figure 3, the nanoparticle tracking analysis (NTA) performed to characterize the size of the sEVs separated by the different approaches does not show changes on size range and concentration induced by DNase I. In the case of DNA-directed immunoprecipitation (IP), the low concentration of particles per milliliter is due to an intrinsic limitation of the method. The number of extracellular vesicles that can be recovered depends on the total surface area of the magnetic beads and on the density of antibodies bound to the surface. On the other hand, the low yield is counterbalanced by the higher purity enabled by the use of sEV-specific antibodies.

### 2.3. TEM Analysis on HEK Cells’ sEVs

**Figure 4:**
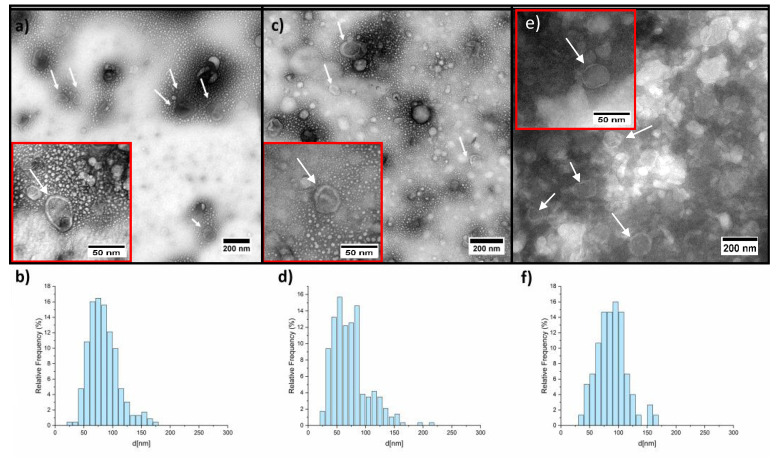
TEM images and histogram of the relative frequency of the EV diameters. Sample: (**a**,**b**) untreated sEVs; (**c**,**d**) sEVs subjected to thermal treatment; and (**e**,**f**) sEVs subjected to DNase I treatment. White arrows indicate isolated sEVs. The boxes represent enlarged sections of the images.

Since the DNase I acts only outside the vesicles, we checked for the integrity of the membrane to exclude a potential rupture induced by the different purification steps. The sEVs isolated from *HEK* cells’ supernatant were submitted to different treatments (with or without DNase I and thermal treatment) and analyzed by TEM. We used extracellular vesicles derived from HEK cells as a model to prove that the membrane of sEVs submitted to DNase I treatment and inactivation remains intact. EVs from HEK cells are from a sample that is cleaner than the plasma, allowing us to avoid artefacts and contaminations. In the presence of membrane rupture, DNase I, if not correctly inactivated, could degrade the DNA inside the vesicles. The images corresponding to the collected sEVs, untreated, after thermal treatment, and after treatment with the DNase I are reported in Figure 4.

From the TEM characterization, it is evident that the EV morphology was not influenced either by the thermal or by the enzymatic treatments. The size distribution does not change significantly, as reported in Table 4. There is no evidence that the thermal and the DNase I treatment influences the sEVs morphology; size range and shape are identical in the two samples. We also estimated the number of sEVs (N_EV_) per µm^2^ to assess if the treatments might have caused a significant reduction in intact sEVs. We analyzed by TEM suspensions of identical initial EV concentration. We calculate the EV density (N_EV_/µm) on the TEM grid for each sample obtaining values of 5.1 for the untreated sEVs, 5.2 for thermal treated sEVs and 3.4 N_EV_/µm for the sEVs treated with DNase I. Considering that in the last case, the presence of EV agglomerate made the counting process difficult, we can assert that neither the thermal treatment nor the DNase I treatment had a dramatic effect on the EV number.

## 3. Discussion

sEVs are increasingly used as biomarkers in several types of cancer due to their functional roles in tumorigenesis, metastasis, and invasion [18]. Exosomes can be taken up by neighboring or distant cells and thereby modulate the function of recipient cells playing a key role in the disease progression. Therefore, they are able to transfer specific signals from the parental cell of origin to the surrounding cells in the microenvironment and to distant organs through the circulatory and lymphatic stream. The exosomes carry functionally active biological material, such as proteins, messenger RNA (mRNAs), and microRNA (miRNAs). In particular, exosome-encapsulated miRNAs are being suggested as novel diagnostic and predictive markers in colorectal cancer [19]. There is a growing body of evidence showing the prognostic and diagnostic value of some exosomal microRNAs in colon cancer (e.g., miR-150, miR-21, miR-192, let-7a, miR-223, and miR-23a) [20]. Moreover, miRNAs were found to be useful in colorectal cancer surgery success prediction: the expression of miR-200 and miR-14 in CRC resected patients’ blood exosomes correlated with the overall survival rate [20]. These findings provide new insights into the application of exosomes as novel non-invasive biomarkers for early detection and risk assessment of patients with colorectal cancer.

Recently, it was also reported that plasma isolation of sEVs improves the detection of mutant DNA in metastatic patients [13]. Combining miRNAs analysis with DNA somatic mutation assessment could give to the physician more complete information concerning the progression of the disease. In the management of mCRC patients, it is crucial to specifically identify *RAS* and *BRAF* mutational status to assess the most useful therapeutic treatment. It is widely recognized that genotyping ctDNA in the plasma of cancer patients is a promising practice to evaluate somatic mutations from solid tumors in a non-invasive way. The role of exosome-associated DNA is still debated. Furthermore, in the literature, there is no consensus on the presence of DNA inside the vesicles yet, and the topic is still open for discussion.

In this work, we analyzed by DNA microarray and ddPCR the sEV-associated DNA in the plasma of eleven mCRC patients and compared the results obtained with those reported in a previous analysis on the ctDNA fraction. In all samples, we found the expected mutation with a fractional abundance often lower compared to that retrieved in ctDNA. However, an unexpected result emerged during the study: in a sample out of eleven, we found a mutation that was not previously identified either in ctDNA or in tissue biopsy. This result could suggest that minimally represented mutations could be below the limit of detection of the methodologies used for the analysis of ctDNA. However, enriching the sEV fractions would allow also revealing mutations present in the circulation at low concentration.

The results of our investigation demonstrate that sEV-associated DNA in plasma can be used as a DNA source for *KRAS* and *BRAF* mutations detection in mCRC patients. The mutations detected in both DNA sources also coincide in samples with a low fractional abundance. Moreover, sEV-associated DNA represents an enriched source of tumoral DNA providing more complete information on all the mutations present in the plasma of a tumor patient, as in the case of one of the samples investigated in our study.

However, the findings on the DNA fraction inside sEVs are less conclusive. It would be extremely important to unequivocally demonstrate that the DNA exists in two forms: externally associated to sEVs, and as cargo. This because DNA inside an EV could have a different role due to its active release from cells instead of a release by apoptosis or necrosis.

To determine if the sEV-associated DNA was outside or inside to the vesicles and to exclude contamination from the ctDNA fraction, we treated one of the samples (sample n.2, see Table 3) with DNase I.

We observed that the amount sEV-associated DNA found after DNase I treatment was, in the best case, only 10% of DNA present before the treatment.

We exclude the possibility that DNase could have been taken up by EVs and cleaved most of the inner DNA.

Proteins such as DNase I cannot passively diffuse across the membrane of sEVs due to their size and polarity unless there are areas of discontinuity in the membrane due to a rupture. The integrity of the membrane of sEVs submitted to a DNAse treatment is clearly demonstrated by the TEM analysis. The behavior of sEVs membrane is similar to that of the cellular membrane. In order to cross a membrane, an active up-take of the protein from the exterior to the interior of the vesicle is needed. We are not aware of the existence of such an active transport mechanism for DNase.

Concerning the existence of a residual activity of DNase, the enzyme was inactivated by adding 5 μL of RQ1 DNase I Stop Solution and samples were heated at 65 °C for 10 min before further analysis.

Our results seem to support the hypothesis that DNA is indeed part of the cargo, but since its amount is only a small fraction of the total sEVs associated amount, it may go undetected, being below the lowest limit of detection of the methods used for its analysis. Even when a small amount is revealed, it is difficult to detect the presence of a mutation. NTA and TEM analyses demonstrated that DNase I and thermal treatments do not impact the number, size, and morphology of separated sEVs. Since the membrane of extracellular vesicles remains intact, the included dsDNA is not affected by enzymatic digestion and can be further analyzed by ddPCR. Despite its small quantity, internal DNA can be effectively distinguished from external DNA (both circulating and membrane-associated DNA). When the ctDNA concentration and the mutation fractional abundance are high in plasma and the method used for the mutational analysis is extremely sensitive, as in the case of ddPCR, it is possible to discriminate two fractions of sEV-associated DNA (sample n.22, see Table 2 DNA-directed immunoprecipitation method with the DNase I treatment entailed in the method).

Our study suggests using both ctDNA and sEV-associated DNA fractions, without DNase I treatment, to identify and monitoring somatic mutations in mCRC patients. We believe that integrating ctDNA and sEV-associated DNA in mCRC patient management could provide a complete real-time assessment of the cancer mutation status. The analysis of DNA cargo is more difficult. The availability of highly sensitive analytical tools would allow to characterize also sEVs’ internal DNA, providing new insights on the role of this marker.

## 4. Materials and Methods

### 4.1. Samples

We collected 12 mL of peripheral blood in EDTA vacutainer tubes from eleven mCRC patients previously genotyped on tissue biopsy at the diagnosis for medication planning followed at the Department of Oncology at San Raffaele Hospital in Milan. The plasma fraction was immediately separated and aliquots of 500 µL of plasma in different vials (about 10–11 for blood sample) were stored at −80 °C until successive analysis. We avoided thawing the same sample several times and used only the plasma volume necessary for each analysis, leaving the other aliquots untouched at −80 °C. All subjects gave their informed consent for inclusion before they participated in the study. The study was conducted in accordance with the Declaration of Helsinki, and the protocol was approved by the Institutional Review Board of the San Raffaele Hospital (ctDNA/2017). The clinical data were collected and previously reported [14].

### 4.2. Isolation of sEVs from Plasma

#### 4.2.1. Ultracentrifugation

One milliliter of plasma (or 250 μL for the experiment with the DNase I treatment) was diluted 1:1 with PBS, and then was filtered with 0.22 mm filters (Merck Millipore, Burlington, MA, USA) and centrifuged in a Optima™ TLX Preparative Ultracentrifuge, Beckman Coulter^TM^ at 150,000× *g* for 120 min at 4 °C with a TLA-55 Rotor (Beckman Coulter^TM^, Brea, CA, USA) to pellet sEVs, mostly exosomes. After careful removal of the supernatant, the sEV-containing pellet was resuspended with PBS (50 μL) and the DNA was extracted.

#### 4.2.2. Exosome Precipitation Kit

Two hundred and fifty microliters of plasma were diluted 1:1 with PBS then filtered with 0.22 mm filters (Merck Millipore, Burlington, MA, USA). Subsequently, the exosomes were isolated using the kit “Exosome Precipitation Solution” by Macherey-Nagel according to the manufacturer’s instructions.

#### 4.2.3. Immunoprecipitation of sEVs on Magnetic Beads Decorated with DNA-Directed AntiCD63

sEVs were immuno-captured on DNA-directed antiCD63. Immobilization of antibodies through a DNA-linker improves the capture efficiency, increasing the probe flexibility. Additionally, the DNA linker can be cleaved using DNase I, enabling the release of extracellular vesicles under mild conditions. The synthesis of ssDNA-antiCD63 conjugate and the DNA sequences used for immunoprecipitation of sEVs are described in [17].

##### Magnetic Beads Functionalization

DNA tagged antibodies were immobilized on the surface of magnetic beads and used to immunocapture sEVs. Prior to their use, streptavidin-coated magnetic beads (Dynabeads M-270 Streptavidin, Invitrogen, Waltham, MA, USA) were washed three times with binding and washing buffer (5 mM Tris-HCl, pH 7.5; 0.5 mM EDTA; 1 M NaCl) according to the manufacturer’s protocol. Then, 500 µg of beads were added to 100 µL of 1µM biotinylated ssDNA-Probe solution. The suspension was stirred for 30 min at 23 °C, then the solution was removed, and the beads were washed twice with B&W buffer, then once with PBS.

Oligonucleotide modified beads (500 µg) were incubated with 100 µL of ssDNA-antiCD63 antibody (160 µg/mL) for 1h at 25 °C, then the solution was removed, and the beads were washed twice in PBS.

##### Capture and Release of sEVs from Magnetic Beads

DNA-directed ssDNA-antiCD63 functionalized magnetic beads (500 µg) were incubated with 250 µL of plasma. After 2.5 h of incubation at room temperature under stirring, the supernatant was removed, and the beads were washed twice with PBS. Then beads were incubated with 50 µL of 250 mKunitz/µL solution of DNase I from bovine pancreas in 10 mM Tris/HCl, pH 7.5 buffer, containing 5 mM MgCl_2_ and 130 µM CaCl_2_. At the end of the incubation, which was performed at 37 °C for 1 h under stirring, the beads were separated using a magnetic stand and the buffer was recovered and used in subsequent analysis.

### 4.3. Extraction of DNA from sEVs and DNase I Treatment

Exosomal DNA was extracted using the Maxwell RSC instrumentation (Promega, Madison, WI, USA) combined with the Maxwell^®^ RSC ccfDNA Plasma Kit (AS1480) and eluted with 60 μL of Elution Buffer.

For the experiment performed with DNAse treatment, 250 μL of plasma were used for the isolation of sEVs, as reported above. EV pellets was resuspended in a digestion mix containing 40 μL of PBS, 5 μL of RQ1 RNase-free DNase I, and 5 μL of RQ1 RNase-free DNase I Reaction Buffer (Promega Corporation, Madison, WI, USA), and incubated for 30′ at 37 °C. After incubation, the DNase I activity was blocked by adding 5 μL of RQ1 DNase I Stop Solution and samples were heated at 65 °C for 10 min to inactivate the enzyme before further analysis.

### 4.4. sEV-Associated DNA Analysis

#### 4.4.1. Microarray Analysis

The DNA sequences encompassing the most frequent mutations of *KRAS* codon 12, 13, 146 and *BRAF* codon 600, were amplified using 5′-biotin forward and 5′-tagged reverse primers. The PCRs were performed in 50 µL solutions containing 15 µL of DNA extracted from purified exosomes, 200 µM deoxynucleotide triphosphates, 10 mM Tris–HCl (pH 8.3), 50 mM KCl, 1.5 mM MgCl_2_, 1.3 U of DNA polymerase (FastStart Taq, Roche, Basel, Switzerland) and 20 pmoles of each primer.

Cycling conditions were as follows: 95 °C for 4 min; 47 cycles at 95 °C for 30 s, 58 °C for 30 s, 72 °C for 30 s and finally 72 °C for 10 min.

Detailed descriptions of the silicon chips coating, the preparation of the microarray and the hybridization assay along with image-scanning and data analysis steps were previously provided in [16].

#### 4.4.2. ddPCR Analysis

We employed the QX100™ Droplet Digital™ PCR System (Bio-Rad Laboratories, Hercules, CA, USA). Eight microliters of eluted exosomal DNA were mixed with primers and fluorophore labeled commercial probes (FAM for the mutated allele and HEX for the wild-type allele) specific for each of the mutations analyzed as previously reported [14]. The fractional abundance of the mutated allele was calculated automatically by the QuantaSoft™ software version 1.7.4 (Bio-Rad), pooling results from the six-fold and deriving fractional abundance of each mutation from a Poisson distribution.

### 4.5. Cell Culture

Human embryonic kidney HEK293 cells were cultured in DMEM (Gibco, Life Technologies Corporation, Grand Island, NY, USA) supplemented with 10% FBS, 2 mM L-glutamine and antibiotics (100 U/mL penicillin and 100 μg/mL streptomycine-sulphate). Cells were grown in incubation at 37 °C under 5% CO2 for maintenance.

#### Exosome Isolation and Treatment with and without DNase I

Three-day-conditioned media from HEK cells cultured in exosome-depleted medium were harvested and centrifuged at 300 g for 25 min. Supernatants were filtered with 0.22 µm filters (Merck Millipore, Burlington, MA, USA) and centrifuged in a Sorvall^TM^ WX Ultracentrifuge (ThermoFisher Scientific, Waltham, MA, USA, WX Ultra 100 #75,000,100) at 150,000× *g* for 2 h at 4 °C with a SureSpin^TM^ 630 swinging bucket rotor (ThermoFisher Scientific) to pellet EV. After supernatant was carefully removed, the EV-containing pellet was resuspended in PBS and stored at −80 °C until use.

The isolated sEVs were aliquoted and submitted to three different protocols. Thirty micrograms of sEVs were treated with 1 μL of DNase I, 1 μL of reaction buffer and then incubated and heated as described above for the DNase I treatment procedure. As a control, thirty micrograms of sEVs were treated in same way, excepted the supplementation of DNase I and heated at 65 °C for 10 min to consider the thermal effect on isolated sEVs (thermal treatment), while the last aliquot of exosomes remained untreated and used as control.

### 4.6. sEVs Analysis

#### 4.6.1. Western Blotting Analysis

Isolated EVs were resuspended in non-reducing Laemmli buffer and boiled for 5 min at 95 °C. Proteins were resolved by SDS-PAGE on the basis of an equivalent quantity of proteins per lane and electrotransferred onto a nitrocellulose membrane. Nonspecific sites were blocked with 5% (*w*/*v*) skimmed milk in T-TBS (tris-buffered saline: 150 mM NaCl, 20 mM TrisHCl, pH 7.4, 0.5% Tween 20).

Membranes were incubated overnight at 4 °C with mouse anti-CD9 (1:2000, BD Pharmingen, #555,370, San Jose, CA, USA) and mouse anti-CD63 (1:10,000; BD Pharmingen, #556,019, San Jose, CA, USA), After washing with T-TBS, membranes were incubated with goat anti-mouse (1:10,000–1:50,000) IgG conjugated to horse-radish peroxidase for 1 h. Positive immunoreactive bands were detected by the enhanced chemiluminescence method (ImmobilonTM HRP substrate, #WBKLS0500, Millipore Corp., Billerica, MA, USA).

#### 4.6.2. Nanoparticle Tracking Analysis (NTA)

sEV samples obtained with different separation approaches were analyzed using Nanosight NS300 (Malvern Panalytical, Malvern, UK). Videos were analyzed by the in-built NanoSight Software NTA 3.2 Dev Build 3.2.16. The camera type, camera level, and detection threshold were sCMOS, 14, and 4, respectively. The number of completed tracks in NTA measurements was 5 (a 60 s movie was registered for each measurement). Sample was diluted in PBS to a final volume of 1 mL. The ideal concentration was assessed by pre-testing the optimal particle per frame value (20–100 particles per frame).

#### 4.6.3. Transmission Electron Microscope (TEM)

The TEM images were collected through ZEISS Libra 200 FE 200 kV equipped with Omega filter in column. The samples were prepared by dropping the EV suspension on a TEM grid covered with formvar/carbon film. After blotting with filter paper, the samples were negative stained using UranyLess (EMS-Electron Microscopy Science) [21]. The EV size was measured by iTEM Imaging platform (Olympus).

## Figures and Tables

**Table 1:** Comparison of results obtained analyzing small extracellular vesicle (sEV)-associated DNA and ctDNA.

	ctDNA *		sEV-Associated DNA	
ID Samples	Microarray	ddPCR Mutated Allele %	Microarray	ddPCR Mutated Allele %
2	*BRAF* V600E	*BRAF* V600E = 15%	*BRAF* V600E	*BRAF* V600E = 4.5%
3	wt		wt	wt
4	wt		wt	wt
7	*KRAS* G12C	*KRAS* G12C = 32.3%	KRAS G12C	*KRAS* G12C = 33.2%
13	*KRAS* A146T	*KRAS* A146T = 20%	*KRAS* A146T	*KRAS* A146T = 13%
14	*KRAS* G13D	*KRAS* G13D = 1.3%	*KRAS* G13D *KRAS* G12D	*KRAS* G13D = 0.35% *KRAS* G12D = na
16	*KRAS* A146T	*KRAS* A146T = 77.5%	*KRAS* A146T	*KRAS* A146T = 77.2%
18	*KRAS* G12D	*KRAS* G12D = 65.3%	*KRAS* G12D	*KRAS* G12D = 64.2%
19	*KRAS* G12S	*KRAS* G12S = 17.7%	*KRAS* G12S	*KRAS* G12S = 16.5%
20	*KRAS* G12D	*KRAS* G12D = 3.7%	*KRAS* G12D	*KRAS* G12D = 4.4%
22	*KRAS* G12D	*KRAS* G12D = 79.8%	*KRAS* G12D	*KRAS* G12D = 80%

* Data previously reported (11), na = not applicable due to sample exhaustion.

**Table 2:** Results obtained by ddPCR analysis for the *KRAS* G12D mutation on ctDNA and sEVs associated DNA.

Patient n. 22	ddPCR Copies/20 µL	Fractional Abundance (Range)	Plasma Volume
ctDNA	mut = 1876 wt = 472 tot = 2348	80% (79–81%)	500 µL
**Isolation of EV**			
Ultracentrifugation	mut = 224 wt = 70 tot = 294	76% (73–79%)	250 µL
Exosome Precipitation Kit	mut = 452 wt = 122 tot = 574	79% (77–81%)	250 µL
DNA-directed Immunoprecipitation *	mut = 7 wt = 14 tot = 22	33% (14–46%)	250 µL

Mut = copies of the mutated allele; wt = copies of the wild-type allele; tot = copies of both mutated and wild-type allele. * DNase I treatment entailed in the method.

**Table 3:** Results obtained by ddPCR analysis for the *BRAF* V600E mutation on ctDNA and sEVs associated DNA with and without DNase I treatment.

Patient n. 2	ddPCR Copies/20 µL	Fractional Abundance (Range)	Plasma Volume
ctDNA	mut = 42 wt = 240 tot = 282	15% (8–21%)	500 µL
**Isolation of EV**			
Ultracentrifugation	mut = 2 wt = 21 tot = 23	8% (2–15%)	250 µL
Ultracentrifugation + DNase I treatment	mut = 0 wt = 2 tot = 2	0	250 µL
Supernatant 1	mut = 15 wt = 92 tot = 107	14% (10–18%)	250 µL
Supernatant 2	mut = 134 wt = 74 tot = 878	15% (10–20%)	250 µL
Exosome Precipitation Kit	mut = 10 wt = 45 tot = 55	19% (12–25%)	250 µL
Exosome Precipitation Kit + DNase I treatment	mut = 0 wt = 0 tot = 0	0	250 µL
DNA-directed Immunoprecipitation *	mut = 0 wt = 3 tot = 3	0	250 µL

Mut = copies of the mutated allele; wt = copies of the wild-type allele; tot = copies of both mutated and wild-type allele. * DNase I treatment entailed in the method.

**Table 4:** Size distribution of sEVs.

**Sample**	d * Mean [nm]	**Std Dev [nm]**	**Minimum [nm]**	**Maximum [nm]**
sEVs untreated	84.5	28.2	28.6	173.7
sEVs thermal treated	74	34.2	24.4	214.7
sEVs treated with DNase I	88.7	26.1	36.2	169.4

* d = diameter.

## Data Availability

Data is contained within the article.
